# Reconstruction of a Complex Foot Defect with a Chimeric Triple-Component Osteocutaneous SCIP–SIEA Free Flap: A Case Report and Literature Review

**DOI:** 10.1055/a-2635-2680

**Published:** 2025-09-01

**Authors:** Yanis Berkane, Elise Lupon, Pierre Muret, Jérôme Laloze, Nicolas Bertheuil, Christian Herlin, Paul Girard, Hadrien Paoli

**Affiliations:** 1 Department of Plastic, Reconstructive and Aesthetic Surgery, Centre Hospitalier Universitaire de Rennes, University of Rennes, Rennes, France; 2 SITI Laboratory, UMR1236, Etablissement Français du Sang, University of Rennes, Rennes, France; 3 Department of Plastic and Reconstructive Surgery, Institut Universitaire Locomoteur et du Sport, Pasteur 2 Hospital, University Côte d'Azur, Nice, France; 4 Department of Orthopaedic Surgery, Centre Hospitalier Universitaire Lapeyronie, University of Montpellier, Montpellier, France; 5 Department of Maxillo-Facial, Plastic and Reconstructive Surgery, Dupuytren University Hospital, Limoges, France; 6 Department of Plastic Reconstructive Surgery and Burns, Centre Hospitalier Universitaire Lapeyronie, University of Montpellier, Montpellier, France; 7 Laboratory of Molecular PhysioMedicine (LP2M), UMR 7370, CNRS, University Côte d'Azur, Nice, France

**Keywords:** foot reconstruction, SCIP flap, chimeric flap, ankle reconstruction, SIEA flap

## Abstract

Complex defects resulting from infected bone or joints with or without osteosynthesis or prosthetic material lead to significant challenges that need to be addressed through orthoplastic approaches. Foot and ankle reconstruction is particularly difficult due to the lack of local or regional flap solutions in this highly mobile joint, which often necessitates microsurgical flaps in extended defects. In addition, rigorous bone reconstruction is critical to acute bone infection to minimize the risks of functional impairments. We present a novel approach using a chimeric osteocutaneous flap to address a complex calcaneus fracture with extended postoperative skin necrosis and septic pseudoarthrosis. A dual skin paddle (16 × 6.5 cm and 14 × 4.5 cm) was created using a superficial inferior epigastric artery (SIEA)-to-superficial circumflex iliac artery (SCIA) anastomosis, while the 7-cm pedicle was increased using a deep inferior epigastric artery graft, which was anastomosed to the tibial anterior vessels. The vascularized iliac crest component enabled optimal reconstruction of the bone defect with rapid healing, while the combined SCIP (superficial circumflex iliac artery perforator)–SIEA skin flap was used to cover the bone reconstruction and skin defect. This microsurgical reconstruction allowed optimal functional recovery at 12 months with successful bone integration and soft tissue coverage. The step-by-step intraoperative technique is described through Video 1 and Supplementary Video 2 .

## Introduction

Acute infection is a severe complication following trauma surgery, with potentially severe functional impairment. Conventional treatments involve surgical debridement of the fracture site, adapted antibiotherapy, interposition of bone grafts, and optimized soft tissue coverage when needed. These techniques can lead to up to 15% failure, 1 2 mostly due to bone graft non-integration. Bone infections are difficult to treat due to the need for high-dose antibiotic delivery to the fracture site, which is challenging with non-vascularized bone tissue such as bone grafts. In case of a primary treatment failure, one relevant technique that can address septic pseudarthrosis treatment is bone flaps, enabling highly vascularized transfers with the resulting ability to fight infection locally. 3 In the lower limb, multioperated, posttraumatic pseudarthrosis is often associated with soft tissue defects that need to be addressed with flap reconstruction. Due to the lack of laxity in this anatomic area, free flaps can be used by the reconstructive surgeon. 4

Since the last decade, the superficial circumflex iliac artery (SCIA) perforator (SCIP) flap has gained increasing interest in the plastic and reconstructive surgery community despite a challenging procurement procedure. 5 6 The SCIP flap provides high-quality anatomic reconstructions with a variety of applications due to its versatility and multiple harvesting techniques. Herein, we propose an innovative use of a triple-component osteocutaneous SCIP–superficial inferior epigastric artery (SIEA) flap to address a complex acute pseudarthrosis of the ankle with severe skin defect. Due to the absence of the inconstant common trunk between these two arteries, a perforator-to-perforator anastomosis was performed between the SCIA and the SIEA. A successful bone reconstruction was achieved by providing a vascularized iliac crest component, while the double skin paddle achieved optimal regloving of the foot and ankle. This procedure, performed on a 32-year-old man, enabled complete ankle healing with successful restoration of its function, with over a year of follow-up.

## Case

**Video 1** Intraoperative view of the procedure (accelerated). The full-length video is available online (See Supplementary Video S2 [available in the online version only]).


**Supplementary Video S1** Video capture of the patient at 18 months postoperatively walking next to his child with full bearing on his right foot. Video courtesy of the patient himself.


**Supplementary Video S2** An extended video capture of the procedure is available at the following link: https://www.youtube.com/watch?v=cn323p_llgA&t=1514s .


All clinical care was compliant with the principles of the Declaration of Helsinki (1964) and the French Bioethics Law (2011). Informed consent was obtained from the patient for the use of his medical record, photographs, and videos.

**Fig. 1:**
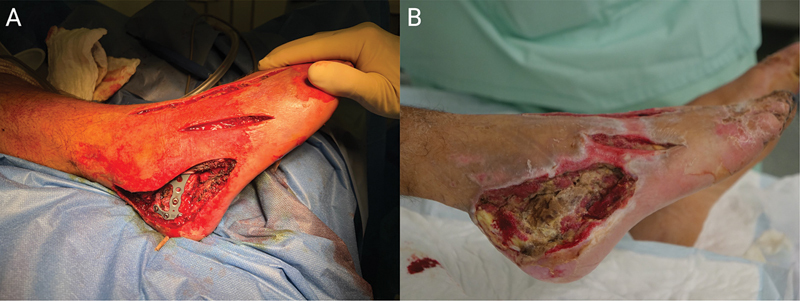
Immediate intraoperative **(A)** and after 12 days **(B)** photographs of the resulting defect following necrosectomy and relief incisions, displaying osteosynthesis material and extended bone exposure and necrosis.

**Fig. 2:**
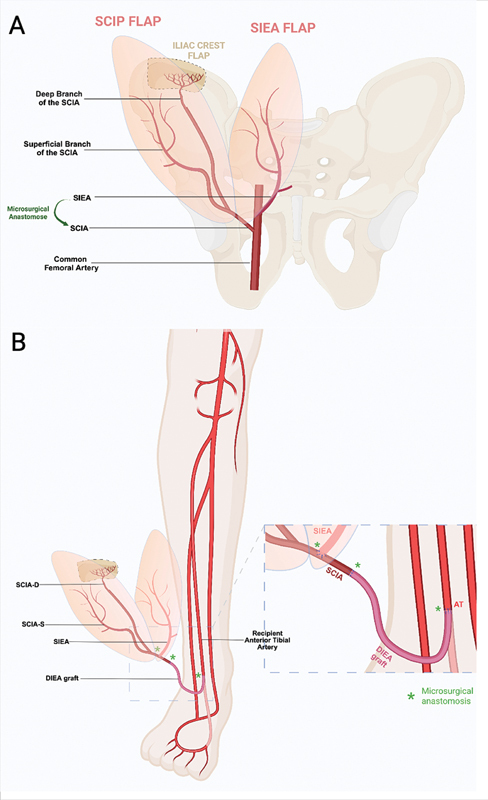
Anatomic scheme displaying the three components of the flap **(A)** . The SIEA pedicle was anastomosed to the SCIA vessels. An iliac crest component was harvested on the deep branch of the SCIA. **(B)** A deep inferior epigastric pedicle graft was used to increase the flap's pedicle length to reach the anterior tibial vessels. All microsurgical anastomoses are displayed. Veins are not displayed to improve clarity, but all the *comitans* veins were anastomosed similarly to the arterial tree. SCIA, superficial circumflex iliac artery; SCIP, superficial circumflex iliac artery perforator; SIEA, superficial inferior epigastric artery.

The patient was a 31-year-old man who suffered from a major trauma on his worksite, resulting in a closed right calcaneus fracture, initially addressed with plate-based osteosynthesis and rapid physiotherapy. Postoperative infection and extended necrotizing fasciitis with *Streptococcus pyogenes* was diagnosed and led to large soft tissue debridement with osteosynthesis material and lateral malleolus exposure, with relief incisions performed ( Fig. 1A ). After 12 days, the patient was discharged from the intensive care unit (ICU) and transferred to the plastic surgery department of our university hospital center ( Fig. 1B ). A multidisciplinary discussion indicated surgical debridement, material removal, bone *curettage* , and immediate reconstruction including bone transfer. A chimeric flap was chosen to achieve vascularized bone transfer, ensuring engraftment and antibacterial distribution to the fracture site. The choice was, therefore, made to perform a chimeric SCIA–SIEA osteocutaneous flap including a right iliac crest component vascularized by the deep SCIA branch ( Fig. 2A ). The SCIP and SIEA skin paddles measured 16 × 6.5 cm and 14 × 4.5 cm, respectively.

**Fig. 3:**
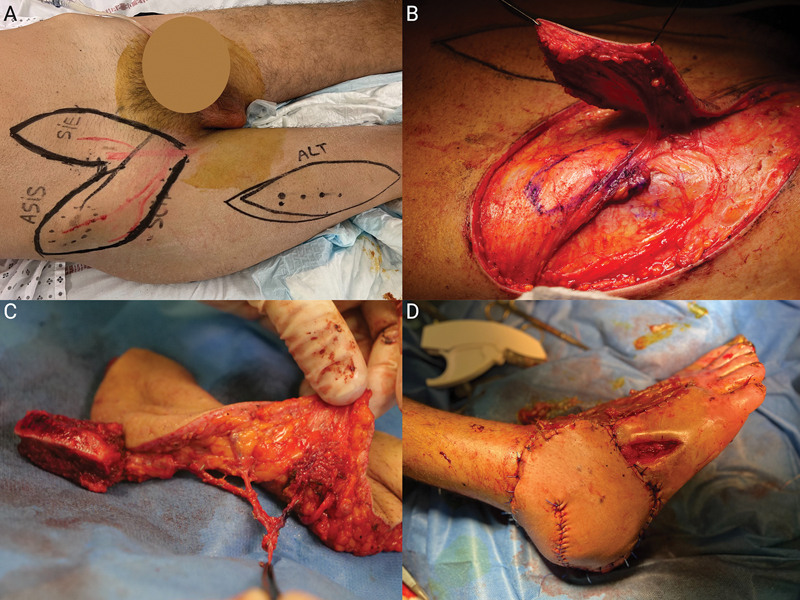
Intraoperative views of the procedure following ultrasound Doppler planning **(A)** , thin SCIP flap procurement on the superficial SCIA branch **(B)** , and SIEA-to-SCIA microsurgical anastomosis achieving three-component chimeric flap building **(C)** . The final aspect **(D)** was obtained after bone impaction, microsurgical anastomoses on the posterior tibial vessels using a vascular bypass based on the deep inferior epigastric vessels. SCIA, superficial circumflex iliac artery; SCIP, superficial circumflex iliac artery perforator; SIEA, superficial inferior epigastric artery.

**Fig. 4:**
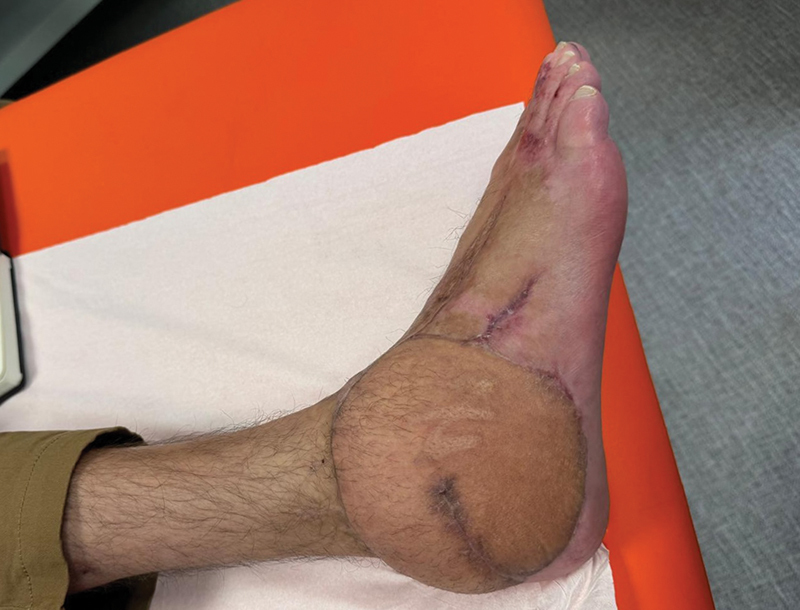
Complete healing obtained after 29 days. The soft tissue coverage obtained with the flap's two skin paddles is moderately bulky.

**Fig. 5:**
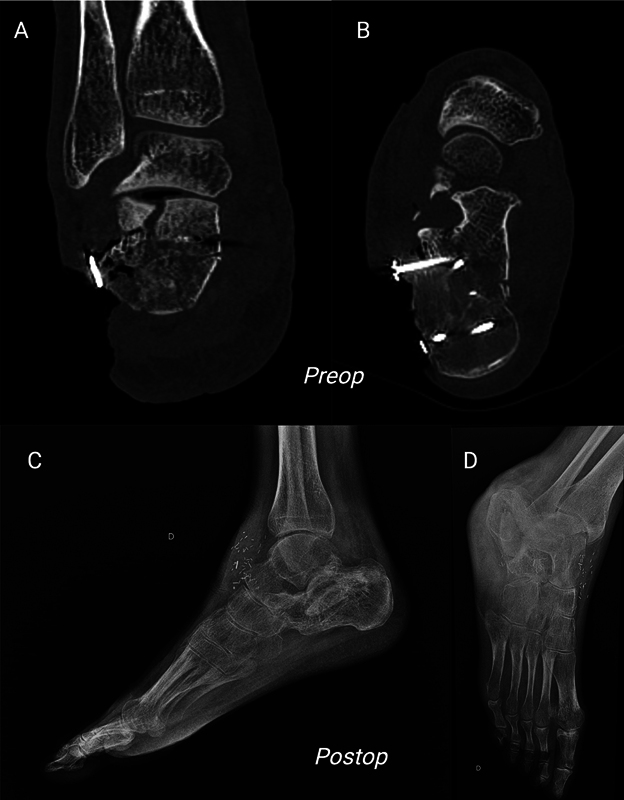
Imaging of the right ankle: Preoperative CT Scan Frontal **(A)** and Transversal **(B)** views. Postoperative X-Ray Medial **(C)** and Lateral **(D)** views, demonstrating optimal integration of the press-fit iliac crest flap into the bone defect. No osteosynthesis material was used to maintain the bone flap.

**Fig. 6:**
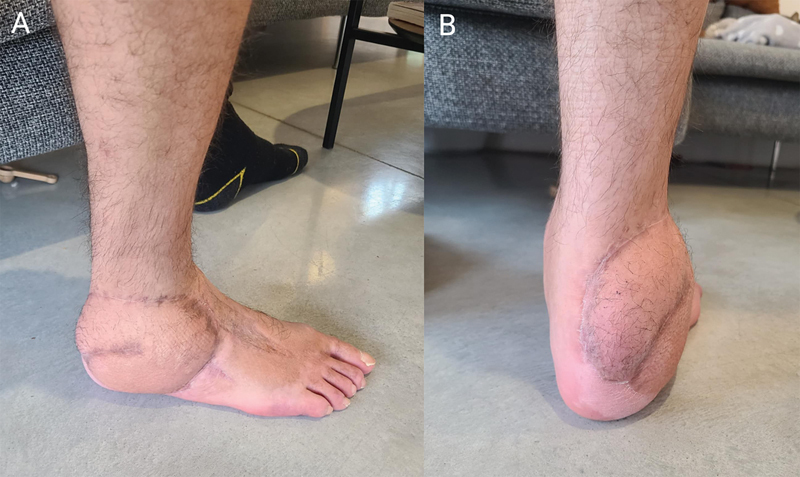
Long-term ouctome (18 months postoperative) with optimal debulking achieved by flap liposuction at 6 months postoperative. Lateral view ( **A** ) and posterior view ( **B** ). The patient can wear regular socks and shoes.

**Fig. 7:**
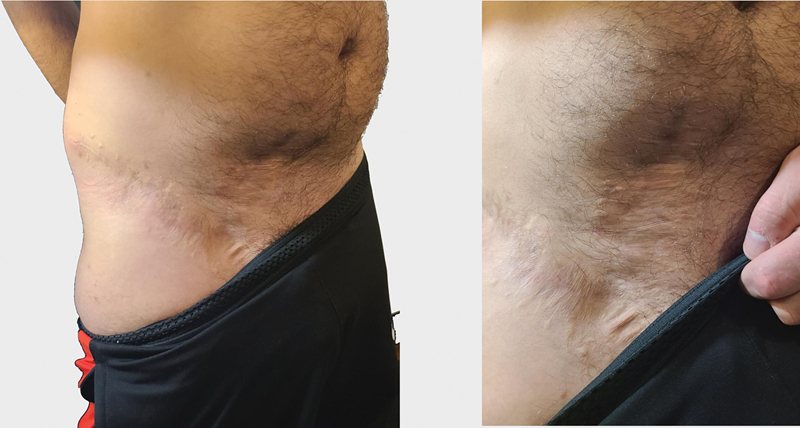
Long-term (18 months postoperative) appearance of the right inguinal donor site.

Preoperative Doppler ultrasound was performed to detect the SCIA perforators, superficial vein, and guide the microvascular dissection. No common SCIA–SIEA trunk was detected on the right vessels (see Supplementary Fig. S1 , available in the online version), while the left side was not considered due to arterial catheterization. Briefly, the SCIA superficial and deep branches were first identified, and a 6 × 2 × 2 cm iliac crest flap was harvested while preserving the periosteum to ensure minimal bone devascularization. The SCIP flap was harvested in the *superficialis fascia* plane ( Fig. 3A , B ), followed by the SIEA flap. A distal-to-proximal pedicle dissection was then pursued until the emergence of the SCIA and SIEA vessels from the common femoral vessels, providing 6- to 7-cm-long pedicles ( Fig. 3C ). An SIEA-to-SCIA perforator-to-perforator anastomosis was performed using 9–0 nylon sutures. A vascular bypass was then performed using deep inferior epigastric vessels to increase the pedicle length and increase the match with the recipient vessel diameter. The triple-component flap was then transferred to the right foot, where the *anterior tibialis* vessels were previously prepared ( Fig. 2B ). End-to-end anastomoses were performed using 9–0 nylon sutures, achieving successful flap revascularization. The iliac crest component was press-fit at the fracture site, with no osteosynthesis to avoid foreign material, and the skin paddle was folded to enable complete coverage of the bone and joint ( Supplementary Video S1 [available in the online version only]). The flap harvesting and intraflap anastomosis and the entire procedure lasted 5 hours 35 minutes and 6 hours 52 minutes, respectively. An artificial dermis (Integra, Princeton, NJ) was used to cover the back of the foot due to the major edema ( Fig. 3D ), followed by a split-thickness skin graft 2 weeks later, achieving complete healing on post operative day 29 ( Fig. 4 ). At 6 months, radiographies demonstrated optimal integration of the iliac crest component ( Fig. 5 ). A moderate liposuction was performed 6 months later to debulk the skin paddles and improve the final cosmetic outcomes and shoe wearing ( Fig. 6 ). No complications were found on the donor site ( Fig. 7 ). The patient underwent biweekly physiotherapy sessions, enabling optimal recovery of ankle mobility and functional walking patterns ( Supplementary Video S1 [available in the online version only]).

## Discussion

In this case, the objective as defined with the patient was limb salvage at any cost, and the risk of limb amputation in case of reconstruction failure was high. Alternative strategies could have involved the absence of a bone flap and soft tissue coverage using a musculocutaneous *latissimus dorsi* (LD) flap. However, this muscle is an important feature for crutching, 7 8 leading to substantial morbidity in case of LD flap failure with amputation and the need for rehabilitation. Moreover, we believe in « Like-for-like » reconstruction, 9 to address each tissue defect more efficiently, therefore indicating a chimeric flap. Chimeric flaps are characterized by several components supplied by a common source vessel. 10 Each component can be mobilized from the others, enabling complex 3D reconstructions. In this case, a chimeric flap using the scapular bone and an LD musculocutaneous flap could have been discussed. However, in our experience, the thoracodorsal artery perforator flap—the perforator version of the LD flap—is less reliable than the SCIP flap. In addition, the skin thickness in the back makes it less adapted for foot and ankle reconstruction, where the native skin is thin. Therefore, the alternative of a muscular LD flap associated with skin grafts is a reliable technique, but it seemed less adapted in this case, in addition to the donor site morbidity. Finally, the cosmetic outcome of such reconstruction would have been unsatisfactory. Another alternative from the back would have been associating a scapular tip flap with a parascapular cutaneous flap, as a chimeric flap. Here again, the dorsal skin seemed less adapted for ankle reconstruction than the inguinal skin. Moreover, the surgery would have required complex positioning in the operative room, and a probable need for multiple position changes, whereas SCIP flaps can be procured in a regular supine position.

Since Koshima et al. in 2004, 11 several authors have described using SCIP flaps in traumatic lower limb reconstruction. Hong and his team increased this flap's popularity through large series, different procurement plane descriptions, and perforator-to-perforator anastomoses. 5 12 13 More specifically, the SCIP flap has been used by several authors for ankle reconstruction in its fasciocutaneous form. Hayashida et al. 14 used a skin paddle based on perforators from the deep branch to cover a posterolateral midsized defect, while Tang et al. 15 described combined SCIP–SIEA flaps for larger defects. If both the SIEA and SCIA vessels can often be used reciprocally, 16 they can also be combined. Suh et al. found a common trunk between both arteries in 10 to 20% of patients, 17 as also confirmed by Fuse et al. in their large retrospective CT scan analysis. 18 If SCIP–SIEA flaps can be valuable flaps for covering large defects, osteocutaneous chimeric flaps can be of greater value. In the groin area, the deep branch of the SCIA dives toward the iliac crest, allowing osteocutaneous flaps based on the SCIA trunk. Vascularized iliac crest transfers based on the SCIA have been widely described and used for various reconstructions, as summarized by Chandra et al. 19 Torrano et al. 20 reported using such a flap for first-ray reconstruction in a radiated foot. They could correct a 3.5-cm bone defect and achieve extensive soft tissue reconstruction with optimal functional outcomes. Similarly, Scharfetter et al. 21 described a chimeric deep SCIA–SIEA flap using the inconsistent common trunk between these two arteries. Yoshimatsu et al. demonstrated pioneering work using chimeric SCIP flaps to address complex ankle defects, including skin-grafted *sartorius* muscle components based on the deep branch. 22 The combination of both chimeric SCIP flaps, including the iliac crest, with a double SCIP–SIEA skin flap remains poorly demonstrated. Scharfetter et al. 21 used an iliac crest flap based on the deep SCIA with a sequential SIEA skin flap to address a complex tibial fracture, optimally addressing both the bone and soft tissue defects. However, the sequential design of their procedure imposed multiple surgeries and exposed them to inflammatory vessel dissection. Another layer of complexity can be added by adding more components, such as vascularized inguinal lymph nodes, portions of the sartorius muscle, and/or vascularized deep fascia, as reported by Yamamoto et al., 23 to address an anterior ankle defect. However, we believe inguinal node dissection should be avoided during SCIP flap procurement for limb reconstruction to avoid risks of lymphedema and/or lymphocele unless preexisting lymphedema needs to be addressed simultaneously.

Yoshimatsu et al. described the intraoperative creation of a chimeric flap using a free fibula covered by a free SCIP skin flap anastomosed to the fibular vessels in a piggyback fashion. 24 This inspired us to perform a triple-component chimeric SCIA–SIEA-based flap created by perforator-to-perforator anastomoses due to the absence of a common trunk between the SIEA and SCIA vessels. To our knowledge, this is the first description of such a setting. A deep inferior epigastric artery graft was decided intraoperatively to increase the pedicle length. Despite the risk of multiplying microsurgical anastomoses, this case demonstrates the possibility of such a complex intraoperative setting in the absence of a common SCIA trunk. The vascularized iliac crest achieved optimal bone reconstruction following acute osteitis, while the bilobed thin flap provided safe and robust soft tissue coverage. An alternative could have been to opt for a larger skin paddle solely based on the SCIA, but vascular safety was preferred. Among the limitations, superthin dissection, as described by Hong et al., 25 26 could have avoided subsequent defatting through liposuction but requires advanced experience and risky dissection.

In the future, developing such advanced reconstructive techniques could lead to reconsidering the indications of bone grafts in complex ankle trauma when trained orthoplastic teams could achieve all-in-one vascularized bone transfers with optimal soft tissue coverage.
