# Supplementary material for: Reconstruction of a Complex Foot Defect with a Chimeric Triple-Component Osteocutaneous SCIP–SIEA Free Flap: A Case Report and Literature Review
Source: Arch Plast Surg. 2025 Sep 1;52(5):310–6. doi: 10.1055/a-2635-2680 (PMC12445957; doi:10.1055/a-2635-2680)
Supplement: Supplementary file 4 — Supplementary Material [file 10-1055-a-2635-2680-s25jan0022cr.pdf]

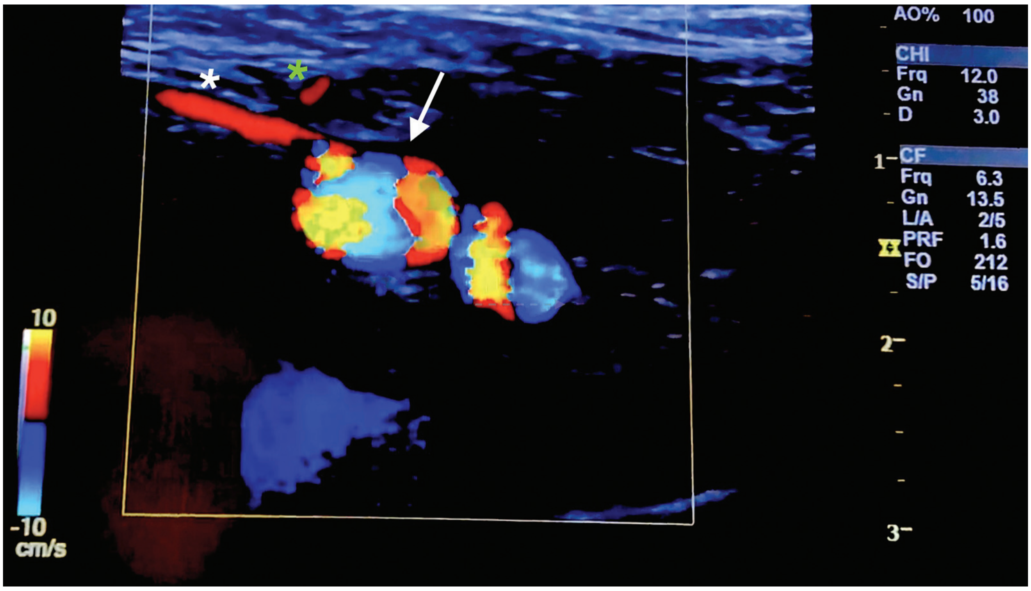


**Supplementary Fig. S1** Preoperative Doppler ultrasound image displaying the origin of the SCIA (white asterisk) emerging from the superficial femoral artery (white arrow) and the absence of a common trunk with the SIEA. SCIA, superficial circumflex iliac artery; SIEA, superficial inferior epigastric artery.

 © 2025. The Author(s).
